# Heterogeneous Data Fusion Method to Estimate Travel Time Distributions in Congested Road Networks

**DOI:** 10.3390/s17122822

**Published:** 2017-12-06

**Authors:** Chaoyang Shi, Bi Yu Chen, William H. K. Lam, Qingquan Li

**Affiliations:** 1 State Key Laboratory of Information Engineering in Surveying, Mapping and Remote Sensing, Wuhan University, Wuhan 430072, China; chaoyangshi@whu.edu.cn; 2 Collaborative Innovation Center of Geospatial Technology, Wuhan 430079, China; 3 Department of Civil and Environmental Engineering, The Hong Kong Polytechnic University, Hong Kong 999077, China; william.lam@polyu.edu.hk; 4 Shenzhen Key Laboratory of Spatial Smart Sensing and Services, Shenzhen University, Shenzhen 518060, China

**Keywords:** travel time distribution, data fusion, evidence theory, spatial correlation, uncertainty

## Abstract

Travel times in congested urban road networks are highly stochastic. Provision of travel time distribution information, including both mean and variance, can be very useful for travelers to make reliable path choice decisions to ensure higher probability of on-time arrival. To this end, a heterogeneous data fusion method is proposed to estimate travel time distributions by fusing heterogeneous data from point and interval detectors. In the proposed method, link travel time distributions are first estimated from point detector observations. The travel time distributions of links without point detectors are imputed based on their spatial correlations with links that have point detectors. The estimated link travel time distributions are then fused with path travel time distributions obtained from the interval detectors using Dempster-Shafer evidence theory. Based on fused path travel time distribution, an optimization technique is further introduced to update link travel time distributions and their spatial correlations. A case study was performed using real-world data from Hong Kong and showed that the proposed method obtained accurate and robust estimations of link and path travel time distributions in congested road networks.

## 1. Introduction

Accurate and robust estimation of travel time distribution, including mean and variance, is a crucial requirement for advanced traveler information systems (ATIS). Provision of travel time distribution information through ATIS enables travelers to make reliable path choice decisions, ensuring a higher chance of on-time arrival [1,2,3]. The provided distribution information also allows operators to evaluate network performance and reliability, and identify bottlenecks for proactively deploying effective controls to improve overall traffic conditions [4,5].

Recent advances in information and communication technologies (ICTs) have produced a variety of spatiotemporal big data for travel time estimation [6]. Existing data collection techniques could be classified into point detection, interval detection, and floating car systems [7,8]. Point detectors (such as loop detectors and video image detectors) are generally deployed at specific road segment locations, to collect vehicle point speeds. Interval detectors consist of a pair of devices deployed in road networks to directly calculate travel times between the device pair. Typical interval detectors include automatic vehicle identification (AVI), Bluetooth, and license plate recognition devices. In contrast to the above fixed detectors, floating car systems use a fleet of probe vehicles, typically taxi cabs, equipped with global positioning system (GPS) devices. The probe vehicle locations and speeds are collected at given time intervals to estimate network traffic conditions [9]. These data collection techniques have generated complementary heterogeneous data sources with distinct data quality and network coverage.

Accurate and robust estimation of travel time distributions from heterogeneous data sources is somewhat challenging in congested road networks. Firstly, although rich traffic observations from multiple data sources are beneficial, data quality variability from different data sources presents a serious impediment to robust estimation of travel time distributions. Data quality variability may raise from a variety of reasons, such as detector failures, measurement errors, sample size variations, etc. [10,11,12,13]. Therefore, traffic observations from different sources can be inconsistent and even conflicting. Thus, robust data fusion techniques are urgently required, relatively insensitive to data quality of heterogeneous data sources.

Secondly, traffic data from multiple data sources has enhanced data availability for major roads in a network, but the limited coverage across the whole network poses a significant challenge to accurate travel time distribution estimates. Traffic data from point detectors cover all vehicles at deployed locations and have a very good temporal sampling, but their spatial coverage is restricted to the (relatively few) deployed locations. Floating car and interval detector data have relatively better spatial coverage on major roads, but sparse data issues remain for many arterial roads [14,15]. Therefore, effective techniques to impute spatially missing data are also required.

This paper proposes an effective method to estimate travel time distributions from heterogeneous data sources with missing data. The remainder of this paper is organized as follows. Section 2 reviews the literature on the travel time distribution estimation methods. Section 3 briefly introduces Dempster-Shafer (D-S) evidence theory to provide the necessary background. Section 4 presents the proposed heterogeneous data fusion method. Section 5 reports a case study using real-world data from Hong Kong. Section 6 provides conclusions and recommendations for further research.

## 2. Literature Review

Travel time estimations have been intensively studied for over three decades. Early studies proposed various methods to estimate deterministic travel times, e.g., mean travel time, using a single data source [16,17,18,19,20]. A complete survey of these methods is beyond the scope of this paper; interested readers can refer to comprehensive reviews by Mori et al. and Vlahogianni et al. [8,21].

In the last decade, many research efforts have focused on data fusion techniques to enhance accuracy and robustness of deterministic travel time estimation using multiple data sources. Current data fusion techniques can be broadly classified into statistical, artificial cognition, and probabilistic-based techniques [22]. Statistical based techniques, such as simple convex combination algorithms, use statistical information of data quality to determine weights for different data sources [16,23]. They are relatively widely used due to their simple implementation. However, they are not well suited to fuse different data sources, which are inconsistent and even conflicting. Artificial cognition based techniques combine multiple data sources using artificial intelligence techniques, such as neural networks or genetic algorithms [11]. They can tackle complex data fusion problems, but require large datasets for training, which are generally infeasible for many real-world applications. Probabilistic based techniques typically employ Bayesian and/or D-S evidence theory to provide mathematical reasoning rules for fusing inaccurate and inconsistent data from multiple sources [24,25,26]. The D-S evidence theory can be regarded as a generalization of Bayesian theory without the requirement of prior knowledge. Nevertheless, most existing studies using D-S evidence theory are restricted to estimation of traffic states (i.e., very congested, congested, medium, smooth or very smooth) rather than precise numerical values of travel times [27,28,29].

Since no single data source covers the whole network, research efforts have also investigated missing data imputing techniques to enhance data completeness. Missing data imputation can be broadly classified as prediction and spatial interpolation based techniques. Prediction-based techniques adopt travel time prediction models, such as K-nearest neighbors, kernel regression, and autoregressive integrated moving average, to forecast the missed data from historical data [30,31,32]. Spatial interpolation techniques impute missing data of a link by using established statistical relationships between the link and its adjacent links [14,15,16,33]. For all techniques in both categories, incorporation of travel time correlations is well recognized as an effective way to improve imputation performance [32]. However, most missing data imputing techniques assume fixed travel time correlations, which are inadequate to represent the dynamic nature of traffic conditions.

The above studies focused on estimating only deterministic travel times, while ignoring travel time variances. Recent attention has investigated methods to estimate travel time distributions (including means and variances) using a single data source. Dion and Rakha [34] proposed an exponential smoothing filter to estimate travel time distributions using interval detector data. Jenelius and Koutsopoulos [35] developed a maximum likelihood approach to estimate travel time distributions using floating car data. Rahmani et al. [36] used the same data type and proposed a nonparametric approach to estimate travel time distributions. Hans et al. [37] used point detector data and proposed a kinematic wave approach for estimating travel time distributions at signalized intersections. Accurate estimation of travel time distributions is more challenging, because more data with higher quality are required to estimate reliable mean and variance information. Including multiple data sources is obviously beneficial for accurate and robust estimations of travel time distributions, but to the best of our knowledge, few studies have been done for estimating travel time distributions by fusing multiple data sources.

To fill this gap, the current study proposes a heterogeneous data fusion method for estimating travel time distributions, fusing interval and point detector data. In the proposed method, link travel time distributions are first estimated from point detector observations. The spatially missing data issue of point detectors is addressed. Travel time distributions of links without point detectors are imputed based on their spatial correlations with links with point detectors. Estimated link travel time distributions from point detector data are then fused with path travel time distributions obtained from interval detectors. To fuse these two path distributions, a D-S distribution fusion algorithm is proposed, built on D-S evidence theory. An optimization technique is further introduced to update link travel time distributions and their spatial correlations according to the fused travel time distribution.

## 3. Brief Introduction of the D-S Evidence Theory

The D-S evidence theory was initially developed by Dempster [38], and later extended and refined by Shafer [39]. This theory can be regarded as a generalization of Bayesian inference to tackle uncertainty reasoning based on incomplete information [40,41]. In contrast to Bayesian inference, D-S evidence theory does not assign priori probabilities to unknown propositions or states [42]. Probabilities are assigned only when supporting evidence is available [43]. This provides a flexible framework for decision making by combining cumulative evidence, and has broad applications in many areas, such as expert systems [40,44], artificial intelligence [45,46], false diagnosis [47,48], target recognition [49,50,51], decision-making [52], information fusion [53], etc.

Let $\Omega =\{S_{1},\ldots ,S_{n}\}$ be a collectively exhaustive and mutually exclusive set of states, which is also called the frame of discernment. This frame of discernment contains every possible state of a system. A basic probability assignment (BPA) (also called a belief structure) is a function $m:2^{\Omega }\to [0,1]$, satisfying m(ϕ)=0 and $\sum_{\forall A\subset \Omega }m(A)=1$, where A is a subset of Ω; and $2^{\Omega }=\{A|A\in \Omega \}$ is the power set of Ω consisting of all the subsets of Ω. The assigned probability m(A) measures the belief exactly assigned to A. All the assigned probabilities sum to unity and there is no belief in the empty set ϕ. For notational consistency, boldfaced letters represent vectors or matrixes throughout the paper.

Multiple independent evidence can be fused using the traditional Dempster’s combination rule [43,44,45,46,47,48,51,52]. With BPAs of two independent evidences, $m_{1}$ and $m_{2}$, the combination rule is defined as:

(1) $$\begin{array}{l} m_{fus}(C)=m_{1}⊕m_{2}=\frac{\sum_{A\cap B=C\neq \phi ,\forall A,B\subset \Omega }m_{1}(A)\times m_{2}(B)}{1-\eta }\\ m_{fus}(\phi )=0 \end{array}$$

where η is the conflict factor, which ranges from 0 to 1 and represents the degree of total conflict between evidences $m_{1}$ and $m_{2}$. 1/(1−η) is the normalization factor which ensures the sum of BPAs can be unit. η is given by:

(2) $$\eta =\sum_{A\cap B=\phi ,\forall A,B\subset \Omega }m_{1}(A)\times m_{2}(B)$$

Dempster’s combination rule, Equation (1), provides effective reasoning rules for fusing low and moderate conflict evidences. However, in case of high or complete conflict evidences (i.e., η value approach to 1), traditional D-S evidence theory may lead to unreasonable synthesis results. To reduce the degree of evidence conflict, an effective method is to modify the evidence. A common technique [54,55] is to introduce an unknown state, Θ, into the frame of discernment as $\Omega^{\prime }=\{\Omega ,\Theta \}$, where Θ represents the unknown part of the evidence.

As an alternative, several researchers argued that high conflict are mainly caused by unreliable evidences; and thereby they proposed methods to identify and correct the unreliable evidences before the combination [48,51,56]. Overall, the D-S evidence theory provides mathematical reasoning rules for fusing inaccurate and incomplete data from multiple sources. In Section 4.2.2, the D-S evidence theory is employed to fuse travel time distributions from different data sources, which may be high conflict or even complete conflict.

## 4. Travel Time Distributions Estimated by Fusing Heterogeneous Data Sources

### 4.1. Problem Statement

Let G=(N,E) be a directed network consisting of a set of nodes, N, and a set of links, E. A link $a_{ij}\in E$ is defined to be the road section between two adjacent nodes with $n_{i}\in N$ and $n_{j}\in N$. Travel time of the link is a random variable, $T_{ij}$, with mean and standard deviation (STD) $t_{ij}$ and $\sigma_{ij}$, respectively. The vector of mean travel times for all links is $t={\left[\ldots ,t_{ij},\ldots \right]}^{T}$, and the variance-covariance matrix between all links is K. The matrix K is the variance-covariance matrix of link travel times. In the variance-covariance matrix K, elements along the diagonal are the variance of link travel times, and off-diagonal elements are the travel time covariance between two links.

Let $p^{od}$ be a path between starting and ending nodes, $n_{o}$ and $n_{d}$, respectively, consisting of λ consecutive links. Let $x_{ij}^{od}$ be a link path incidence variable, where $x_{ij}^{od}=1$ means that link $a_{ij}$ is on $p^{od}$ and $x_{ij}^{od}=0$ otherwise. Let $X={\left[\ldots ,x_{ij}^{od},\ldots \right]}^{T}$ be the vector of link path incidence variables. The path travel time distribution, $T^{od}$, can be calculated by summing link travel times along the path,

(3) $$T^{od}=\sum_{\forall a_{ij}\in E}T_{ij}x_{ij}^{od}$$

Let $t^{od}$ and $\sigma^{od}$ be the mean and variance of the path travel time distribution, respectively, then:

(4) $$t^{od}=X^{T}t$$

(5) $$\sigma^{od}=\sqrt{X^{T}KX}$$

**Figure 1:**
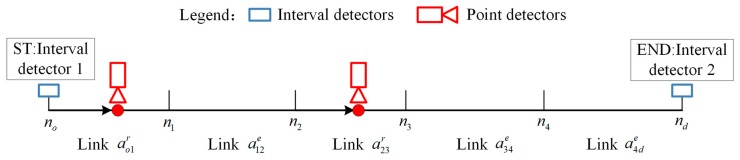
Illustrative example of the heterogeneous data fusion problem.

To obtain travel time distribution information, many detectors of different types may be deployed in the network, as shown in Figure 1 for a simple network with interval and point detectors. A pair of interval detectors, e.g., AVI devices, are installed at $n_{o}$ and $n_{d}$ of $p^{od}$ to record the set of vehicles passing them. The path travel time of each recorded vehicle can be obtained by the time difference from entering to leaving the path, and path travel time distribution can be directly estimated from this data, denoted as $T_{int}^{od}$. However, the detailed travel time distributions of all links along the path are unknown and the interval detector data covers only a portion of vehicles with relatively poor temporal sampling.

Point detectors, e.g., loop detectors, are generally deployed for a subset of network links in real applications, e.g., $a_{o1}^{r}$ and $a_{23}^{r}$ in Figure 1, whereas other links, e.g., $a_{12}^{e}$, $a_{34}^{e}$, and $a_{4d}^{e}$, are without detectors. Thus, only travel time distributions of links with point detectors, e.g., $T_{o1}^{r}$ and $T_{23}^{r}$, can be directly estimated, while travel time distributions of links without point detectors are unknown, e.g., $T_{12}^{e}$, $T_{34}^{e}$, and $T_{4d}^{e}$. Nevertheless, the point detector data tend to have good temporal sampling, since these detectors generally can collect the speeds of all vehicles passing through them.

Obviously, interval and point detector data have distinct spatial and temporal characteristics. Fusing heterogeneous data from both interval and point detectors could be beneficial for estimating travel time distributions for the path and all links either with or without point detectors.

### 4.2. Proposed Heterogeneous Data Fusion Method

This section presents the proposed heterogeneous data fusion method to estimate travel time distributions for the path and all links either with or without point detectors. Empirical studies have found that travel times can be well represented by either normal, gamma, or lognormal distributions [10,39]. Therefore, to simplify the problem and present the essential concept, it is assumed that all link and path travel time distributions follow the normal distribution [57,58]. Using this normality assumption, the proposed method is to estimate the mean and STD of travel time distributions of the path and all links.

**Figure 2:**
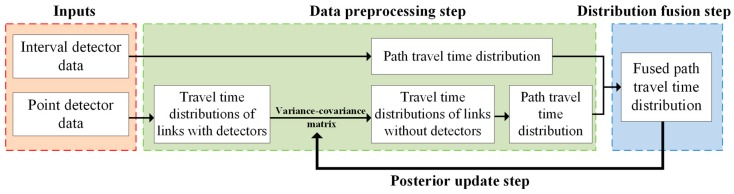
Framework of the proposed heterogeneous data fusion method.

Figure 2 shows that the framework of the proposed heterogeneous data fusion method consists of three steps, described in detail in the following sections. The first step, called data preprocessing, is to estimate path travel time distributions from interval and point detector data, respectively. The second step, called distribution fusion, is to fuse the estimated path travel time distributions by using D-S evidence theory. The last step, called posterior update, is to update link travel time distributions and their travel time correlations based on the fused distribution.

#### 4.2.1. Data Preprocessing Step

This step estimates path travel time distributions from interval and point detector data, independently. The path travel time distribution, $T_{int}^{od}$, can be directly estimated from interval detector data. Since interval detectors only record a set of vehicles equipped with electronic tags, the limited sample size becomes a critical issue in the estimation, especially for low market penetration applications. Outlier observations can also significantly affect path travel time distribution accuracy, e.g., some vehicles may make stops or detours along the path, leading to atypical travel time observations. To estimate path travel time distribution from interval detector data, the data filtering algorithm proposed by Dion and Rakha [34] was adopted in this study. This data filtering algorithm utilizes a series of low pass filters to remove outlier observations outside a dynamically varying validity window. Such algorithms can perform well in both stable and unstable traffic conditions at low levels of market penetration; and have been successfully applied in the real-time traveler information system (RTIS) in Hong Kong [14]. Thus, an accurate and robust estimation of mean, $t_{int}^{od}$ and STD, $\sigma_{int}^{od}$ of path travel time distribution can be obtained from interval detector data.

As discussed above, path travel time distribution cannot be directly estimated through point detector data, because only a few links are deployed with point detectors. To estimate the path travel time distributions, links are divided into links with and without point detectors, so that the vector of mean travel time comprises two parts $t_{\mathrm{poi}}=[t_{\mathrm{poi}}^{r},t_{\mathrm{poi}}^{e}{]}^{T}$, where $t_{\mathrm{poi}}^{r}$ and $t_{\mathrm{poi}}^{e}$ are mean travel times for links with and without point detectors, respectively, at time interval ℓ. The variance-covariance matrix can be divided into four sub-matrixes, $K_{\mathrm{poi}}=\left[\begin{array}{cc} K_{\mathrm{poi}}^{\mathrm{rr}} & K_{\mathrm{poi}}^{\mathrm{er}}\\ K_{\mathrm{poi}}^{\mathrm{re}} & K_{\mathrm{poi}}^{\mathrm{ee}} \end{array}\right]$, where $K_{\mathrm{poi}}^{\mathrm{rr}}$ is the variance-covariance matrix for links with point detectors; $K_{\mathrm{poi}}^{\mathrm{ee}}$ is the variance-covariance matrix for links without point detectors; $K_{\mathrm{poi}}^{\mathrm{er}}$ is the covariance matrix between links without and with point detectors; and $K_{\mathrm{poi}}^{\mathrm{re}}={\left(K_{\mathrm{poi}}^{\mathrm{er}}\right)}^{T}$ is the covariance matrix between links with and without point detectors. Let $v_{\mathrm{poi}}^{r}$ and $v_{\mathrm{poi}}^{e}$ be vectors of travel time variances for links with and without point detectors, respectively. They are elements along the diagonal of $K_{\mathrm{poi}}^{\mathrm{rr}}$ and $K_{\mathrm{poi}}^{\mathrm{ee}}$, respectively.

For a link $a_{r}^{i}$ with a point detector, its mean, $t_{r}^{i}$, and STD, $\sigma_{r}^{i}$, of the link travel time distribution can be obtained from the collected data at the current time interval ℓ, i.e., $t_{\mathrm{poi}}^{r}$ and $K_{\mathrm{poi}}^{\mathrm{rr}}$ can be determined from the point detector data. However, mean travel times for links without point detectors, $t_{\mathrm{poi}}^{e}$, should be indirectly estimated. Following Tam and Lam [14], $t_{\mathrm{poi}}^{e}$ is estimated using spatial correlations between links with and without point detectors:

(6) $$t_{\mathrm{poi}}^{e}=t_{\mathrm{upd}}^{e,\ell -1}+K_{\mathrm{upd}}^{\mathrm{er},\ell -1}{\left(K_{\mathrm{poi}}^{\mathrm{rr}}\right)}^{-1}(t_{\mathrm{poi}}^{r}-t_{\mathrm{upd}}^{r,\ell -1})$$

where $t_{\mathrm{upd}}^{r,\ell -1}$ and $t_{\mathrm{upd}}^{e,\ell -1}$ are mean travel times for the links with and without point detectors estimated at the previous time interval ℓ−1, respectively; $K_{\mathrm{upd}}^{\mathrm{er},\ell -1}$ is the covariance matrix between links without and with point detectors estimated at the previous time interval ℓ−1; and ${\left(K_{\mathrm{poi}}^{\mathrm{rr}}\right)}^{-1}$ is the inverse of $K_{\mathrm{poi}}^{\mathrm{rr}}$.

Similar to Equation (6), $v_{\mathrm{poi}}^{e}$ in this study was also indirectly estimated using the spatial correlations between links with and without point detectors:

(7) $$v_{\mathrm{poi}}^{e}=v_{\mathrm{upd}}^{e,\ell -1}+K_{\mathrm{upd}}^{\mathrm{er},\ell -1}{\left(K_{\mathrm{poi}}^{\mathrm{rr}}\right)}^{-1}(v_{\mathrm{poi}}^{r}-v_{\mathrm{upd}}^{r,\ell -1})$$

where $v_{\mathrm{upd}}^{r,\ell -1}$ and $v_{\mathrm{upd}}^{e,\ell -1}$ are travel time variances of the links with and without point detectors at the previous time interval ℓ−1, respectively. Therefore, elements along the diagonal of $K_{\mathrm{poi}}^{\mathrm{ee}}$ and all elements of $K_{\mathrm{poi}}^{\mathrm{rr}}$ are estimated in the current time interval ℓ. It is assumed that ${\left(k_{poi}^{ee}\right)}_{ij}={\left(k_{upd}^{ee,\ell -1}\right)}_{ij},\forall i\neq j$ and $K_{\mathrm{poi}}^{\mathrm{er}}=K_{\mathrm{upd}}^{\mathrm{er},\ell -1}$, which means that off-diagonal elements of $K_{\mathrm{poi}}^{\mathrm{ee}}$ and all elements of $K_{\mathrm{poi}}^{\mathrm{er}}$ are the same as corresponding elements at the previous interval, ℓ−1. These two matrixes, $K_{\mathrm{poi}}^{\mathrm{ee}}$ and $K_{\mathrm{poi}}^{\mathrm{er}}$, will be updated in the posterior update step in Section 4.2.3.

After $t_{\mathrm{poi}}=[t_{\mathrm{poi}}^{r},t_{\mathrm{poi}}^{e}{]}^{T}$ and $K_{\mathrm{poi}}=\left[\begin{array}{cc} K_{\mathrm{poi}}^{\mathrm{rr}} & K_{\mathrm{poi}}^{\mathrm{er}}\\ K_{\mathrm{poi}}^{\mathrm{re}} & K_{\mathrm{poi}}^{\mathrm{ee}} \end{array}\right]$ are determined, the mean, $t_{poi}^{od}$, and STD, $\sigma_{poi}^{od}$, of the path travel time distribution, can be calculated. The vector of link path incidence variables is divided into two groups as $X=[X_{\mathrm{poi}}^{r},X_{\mathrm{poi}}^{e}{]}^{T}$, where $X_{\mathrm{poi}}^{r}$ and $X_{\mathrm{poi}}^{e}$ are link path incidence variables for links with and without point detectors, respectively. Then, Equations (4)–(7) for calculating $t_{poi}^{od}$ and $\sigma_{poi}^{od}$ can be expressed as:

(8) $$t_{poi}^{od}={\left(X_{\mathrm{poi}}^{r}\right)}^{T}t_{\mathrm{poi}}^{r}+{\left(X_{\mathrm{poi}}^{e}\right)}^{T}t_{\mathrm{poi}}^{e}$$

(9) $$\sigma_{poi}^{od}=\sqrt{{\left(X_{\mathrm{poi}}^{r}\right)}^{T}K_{\mathrm{poi}}^{\mathrm{rr}}X_{\mathrm{poi}}^{r}+{\left(X_{\mathrm{poi}}^{e}\right)}^{T}K_{\mathrm{poi}}^{\mathrm{ee}}X_{\mathrm{poi}}^{e}+2{\left(X_{\mathrm{poi}}^{e}\right)}^{T}K_{\mathrm{poi}}^{\mathrm{er}}X_{\mathrm{poi}}^{r}}$$

Substituting Equation (6) into Equation (8), the mean travel time can be expressed as:

(10) $$t_{poi}^{od}={\left(X_{\mathrm{poi}}^{r}\right)}^{T}t_{\mathrm{poi}}^{r}+{\left(X_{\mathrm{poi}}^{e}\right)}^{T}t_{\mathrm{upd}}^{e,\ell -1}+{\left(X_{\mathrm{poi}}^{e}\right)}^{T}K_{\mathrm{upd}}^{\mathrm{er},\ell -1}{\left(K_{\mathrm{poi}}^{\mathrm{rr}}\right)}^{-1}(t_{\mathrm{poi}}^{r}-t_{\mathrm{upd}}^{r,\ell -1})$$

Therefore, the path travel time distribution, $T_{poi}^{od}$, can be determined from point detector data.

#### 4.2.2. Distribution Fusion Step

This step fuses two path travel time distributions, $T_{int}^{od}$ and $T_{poi}^{od}$, estimated from interval and point detectors, respectively. A fusion algorithm is proposed built on the D-S evidence theory. In this study, the frame of discernment, Ω, is defined as a set of mutually exclusive travel time ranges, $\left\{S_{1},\ldots ,S_{i},\ldots ,S_{n}\right\}$, where each travel time range, $S_{i}=[l_{i},u_{i}]$, is defined by a lower bound $l_{i}$ and upper bound $u_{i}$.The mean travel time for range $S_{i}$ can be expressed as:

(11) $$E(S_{i})=\frac{u_{i}+l_{i}}{2}$$

**Figure 3:**
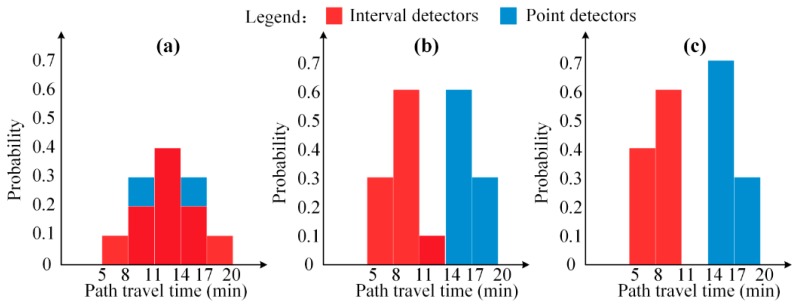
Typical information conflict situations of interval and point detectors: (**a**) low conflict, (**b**) high conflict, (**c**) complete conflict.

Path travel time distributions estimated by interval and point detectors can be regarded as two independent sets of evidence. Based on the defined travel time ranges, these two path travel time distributions are discretized to obtain corresponding discrete distributions, i.e., histograms, as illustrated in Figure 3a. The resultant discrete distributions, $m_{int}$ and $m_{poi}$, are respectively modelled as BPAs for $T_{int}^{od}$ and $T_{poi}^{od}$. Then, $m_{*}(S_{i})$ (either $m_{int}(S_{i})$ or $m_{poi}(S_{i})$) represents the corresponding probability of travel time range *S_i_*, and can be expressed as:

(12) $$m_{*}(S_{i})=\int_{l_{i}}^{u_{i}}f_{*}(x)dx$$

where $f_{*}(x)$ is the probability density function of $T_{*}^{od}$ (either $T_{int}^{od}$ or $T_{poi}^{od}$). When path travel time distributions follow normal distributions, $m_{*}(S_{i})$ can be expressed as:

(13) $$m_{*}(S_{i})=\Phi_{snd}(\frac{u_{i}-t_{*}^{rs}}{\sigma_{*}^{rs}})-\Phi_{snd}(\frac{l_{i}-t_{*}^{rs}}{\sigma_{*}^{rs}})$$

where $\Phi_{snd}(\cdot )$ represents the cumulative distribution function (CDF) of the standard normal distribution. In the literature, Hart’s formula [59] is a good numerical approximation approach to calculate $\Phi_{snd}(\cdot )$:

(14) $$\Phi_{snd}(x)≅\frac{1}{2}-\frac{1}{x\sqrt{2\pi }}\left\{e^{-x^{2}/2}-[\frac{\pi x^{2}}{2}+\frac{{\left(1+0.282455x^{2}\right)}^{1/2}}{1+0.212024x^{2}}e^{-x^{2}/2}]\right\}$$

Clearly, BPAs, $m_{*}$, satisfies $m_{*}(\phi )=0$ and $\sum_{\forall S_{i}\subset \Omega }m_{*}(S_{i})=1$.

Figure 3 illustrates three typical situations of evidence conflict, representing the relationships between interval detector and point detector. Two path travel time are discretized into five travel time ranges as (5, 8), (8, 11), (11, 14), (14, 17) and (17, 20) which constitute the frame of discernment $\Omega =\{S_{1},S_{2},S_{3},S_{4},S_{5}\}$. The corresponding BPAs of two path travel time distributions are shown in Table 1. Figure 3a shows Case 1 that the two evidences have high belief level and low conflict degree, with a large portion of histogram coverage. Figure 3b shows Case 2 that two evidences have low belief level and high conflict degree, with only a small portion of histogram coverage. Figure 3c shows Case 3 that the two evidences are completely conflicted without histogram coverage.

Table 1 shows the results of evidence fusion by using Dempster’s combination rule, Equation (1). As shown, this combination rule can provide a good estimation of path travel time distribution for Case 1 with a low conflict factor, $\eta =1-\sum_{i=1}^{5}m_{int}(S_{i})\times m_{poi}(S_{i})=1-(0.2\times 0.3+0.4\times 0.4+0.2\times 0.3)=0.72$. The fused BPA is calculated from $m_{fus}(S_{i})=m_{int}(S_{i})\times m_{poi}(S_{i})/(1-\eta )$ (e.g., $m_{fus}(S_{3})=0.4\times 0.4/(1-0.72)=0.5714$). After distribution fusion, travel time ranges, $S_{2}$, $S_{3}$ and $S_{4}$, supported by both evidence sets, are strengthened in a reasonable way.

However, for Cases 2 with high conflict factor, η=1−(0.1×0.1)=0.99, the Dempster’s combination rule can lead to an incorrect fusion result, $m_{fus}(S_{3})=(0.1\times 0.1)/(1-0.99)=1$, given both evidence sets afford little support to $S_{3}$. This situation is known as Zadeh’s paradox in the literature. Further, Dempster’s combination rule cannot be used for Case 3 of the completely conflict situation. In this case, $m_{int}(\cdot )$ and $m_{poi}$ cannot be fused, because η=1 so all $m_{fus}(S_{i})$ become infinite.

To reduce the degree of data conflict, the generalized combination rule, Equation (2), is adopted in this study, by introducing the unknown state into the frame of discernment, $\Omega =\{S_{1},\ldots ,S_{i},\ldots ,S_{n},\Theta \}$. Subsequently, to construct BPA $m_{*}$ (either $m_{int}$ or $m_{poi}$), a pre-defined small probability $\alpha_{\Theta }=m_{*}(\Theta )$, (e.g., $\alpha_{\Theta }=0.05$), is set for the unknown state Θ. Then, the path travel time distribution between $t_{*}^{od}+\Phi_{snd}^{-1}(\alpha_{\Theta }/2)\sigma_{*}^{od}$ and $t_{*}^{od}+\Phi_{snd}^{-1}(1-\alpha_{\Theta }/2)\sigma_{*}^{od}$ is discretized to obtain $m_{*}(S_{i})$, where $\Phi_{snd}^{-1}(\cdot )$ is the inverse CDF of the standard normal distribution (e.g., $\Phi_{snd}^{-1}(0.025)=-1.96$ and $\Phi_{snd}^{-1}(0.975)=1.96$).

High and complete conflict situations are usually due to various data quality from the different detectors. To differentiate data sources with varying quality, an information quality parameter [48] is adopted in this study to assign higher weighting to data sources with better information quality. Let $w_{int}$ and $w_{poi}$ be the information quality weights for the path travel time distribution from interval and point detectors respectively. In this study, $w_{int}$ and $w_{poi}$ are expressed as a function of sample size and travel time variance:

(15) $$w_{int}=\frac{1-{\left(1-\beta_{int}\right)}^{N_{int}}}{{\left(\sigma_{int}^{od}\right)}^{2}}$$

(16) $$w_{poi}=\frac{1-{\left(1-\beta_{poi}\right)}^{N_{poi}}}{{\left(\sigma_{poi}^{od}\right)}^{2}}$$

where $N_{int}$ is the sample size collected by interval detectors; $N_{poi}$ is the average sample size for all point detectors along the path; $\beta_{int}$ and $\beta_{poi}$ are sensitivity parameters for interval and point detectors, respectively, which should be calibrated independently. Other types of information quality function could also be used in practice.

Applying different weightings $w_{int}$ and $w_{poi}$, the BPA $m_{*}$ (either $m_{int}$ or $m_{poi}$) is adjusted using following formula [48]:

(17) $${\bar{m}}_{*}=\left\{ \begin{array}{l} {\bar{m}}_{*}(S_{i})=\frac{w_{*}}{w_{\max}}\cdot m_{*}(S_{i}),\forall S_{i}\subseteq \Omega \\ {\bar{m}}_{*}(\Theta )=1-\sum_{i=1}^{n}{\bar{m}}_{*}(S_{i}) \end{array}\right.$$

where $w_{\max}=\max(w_{int},w_{poi})$ is the larger between $w_{int}$ and $w_{poi}$. Substituting the adjusted BPAs into Equations (1) and (2), the fused BPA, $m_{fus}$, can be determined following the generalized combination rule:

(18) $$m_{fus}=\left\{ \begin{array}{l} m_{fus}(S_{i})=\frac{{\bar{m}}_{int}(S_{i})\times {\bar{m}}_{poi}(S_{i})+{\bar{m}}_{int}(S_{i})\times {\bar{m}}_{poi}(\Theta )+{\bar{m}}_{int}(\Theta )\times {\bar{m}}_{poi}(S_{i})}{1-\eta },\forall S_{i}\subseteq \Omega \\ m(\Theta )=\frac{{\bar{m}}_{int}(\Theta )\times {\bar{m}}_{poi}(\Theta )}{1-\eta }\\ \eta =1-{\bar{m}}_{int}(\Theta )\times {\bar{m}}_{poi}(\Theta )-\sum_{i=1}^{n}\left[{\bar{m}}_{int}(S_{i})\times {\bar{m}}_{poi}(S_{i})+{\bar{m}}_{int}(\Theta )\times {\bar{m}}_{poi}(S_{i})+{\bar{m}}_{poi}(\Theta )\times {\bar{m}}_{int}(S_{i}\right) \end{array}\right.$$

Table 2 illustrates the distribution fusion built on the generalized combination rule using the same example as in Table 1. In this example, $m_{int}(\Theta )=m_{poi}(\Theta )=0.05$ are set; and two BPAs, $m_{int}$ and $m_{poi}$ are modified to reflect this setting. Information quality parameters $w_{int}=0.8$ and $w_{poi}=0.6$ are used for interval and point detectors, respectively. All BPAs, $m_{poi}$, for these cases are adjusted to ${\bar{m}}_{poi}(S_{i})=m_{poi}(S_{i})\times 0.6/0.8$, $\forall S_{i}\subset \Omega$; and ${\bar{m}}_{poi}(\Theta )=1-\sum_{i=1}^{5}{\bar{m}}_{poi}(S_{i})=1-0.95\times 0.6/0.8=0.288$. The generalized combination rule, Equation (16), was adopted for fusing path travel time distribution.

Table 2 shows that the generalized combination rule provides a reasonable outcome for Case 1 (i.e., low conflict situation). More importantly, this generalized combination rule can well address the distribution fuse problem for Case 2 (i.e., the high conflict situation). Introducing Θ significantly reduced the conflict factor η to 0.6614. The probability of $S_{3}$, which has little support from both evidence sets, is only slightly strengthened as $m_{fus}(S_{3})=(0.075\times 0.056+0.075\times 0.288+0.05\times 0.056)/(1-0.6614)=0.0874$. The probabilities of other travel time ranges, $S_{1}$, $S_{2}$, $S_{4}$, and $S_{5}$, are reduced, but a higher weighting is given to the data source with better data quality (i.e. $m_{int}$). The generalized combination rule also addressed the distribution fusion for Case 3 (i.e., complete conflict situation), which cannot be fused using Dempster’s combination rule.

From the fused BPA, $m_{fus}$, the corresponding mean and STD, can be expressed as:

(19) $$\theta =\frac{1}{1-m_{fus}(\Theta )}$$

(20) $$t_{fus}^{od}=\sum_{i=1}^{n}\theta \cdot m_{fus}\cdot (S_{i})\cdot E(S_{i})$$

(21) $$\sigma_{fus}^{od}=\sqrt{\sum_{i=1}^{n}{\left(E(S_{i})-t_{fus}^{od}\right)}^{2}\cdot \theta \cdot m_{fus}\cdot (S_{i})}$$

where θ is the adjustment parameter to assign the probability of the unknown state to each travel time range. Thus, the proposed D-S distribution fusion algorithm can estimate path travel time distributions by fusing two path travel time distributions from interval and point detector data, even in the cases of extreme conflict between the data sets.

##### 4.2.3. Posterior Update Step

This step updates the link travel time distributions and their spatial correlations based on the fused path travel time distribution. An optimization technique is proposed to update the travel time means (i.e., $t_{\mathrm{poi}}=[t_{\mathrm{poi}}^{r},t_{\mathrm{poi}}^{e}{]}^{T}$) and variance-covariance matrix (i.e., $K_{\mathrm{poi}}=\left[\begin{array}{cc} K_{\mathrm{poi}}^{\mathrm{rr}} & K_{\mathrm{poi}}^{\mathrm{er}}\\ K_{\mathrm{poi}}^{\mathrm{re}} & K_{\mathrm{poi}}^{\mathrm{ee}} \end{array}\right]$) estimated in the data preprocessing step.

Let $t_{\mathrm{upd}}=[t_{\mathrm{upd}}^{r},t_{\mathrm{upd}}^{e}{]}^{T}$ and $K_{\mathrm{upd}}=\left[\begin{array}{cc} K_{\mathrm{upd}}^{\mathrm{rr}} & K_{\mathrm{upd}}^{\mathrm{er}}\\ K_{\mathrm{upd}}^{\mathrm{re}} & K_{\mathrm{upd}}^{\mathrm{ee}} \end{array}\right]$ be the updated travel time means and covariance matrix, respectively where ${\left(k_{upd}^{ee}\right)}_{ij}$ is the element at row i and column j of $K_{\mathrm{upd}}^{\mathrm{ee}}$. This study uses $t_{\mathrm{upd}}^{r}=t_{\mathrm{poi}}^{r}$ and $K_{\mathrm{upd}}^{\mathrm{rr}}=K_{\mathrm{poi}}^{\mathrm{rr}}$, because $t_{\mathrm{poi}}^{r}$ and $K_{\mathrm{poi}}^{\mathrm{rr}}$ are directly obtained from point detector data and assumed to be accurate. Therefore, to update the link travel time covariance matrix, only $K_{\mathrm{upd}}^{\mathrm{ee}}$ and $K_{\mathrm{upd}}^{\mathrm{er}}$ sub-matrixes need to be updated, since $K_{\mathrm{upd}}^{\mathrm{re}}={\left(K_{\mathrm{upd}}^{\mathrm{er}}\right)}^{T}$ holds. Accordingly, the optimization problem of updating the spatial correlations is formulated as the following nonlinear programming problem:

(22) $$M1\;\min\left(\sum_{\forall i}\sum_{\forall j}{\left({\left(k_{upd}^{ee}\right)}_{ij}-{\left(k_{poi}^{ee}\right)}_{ij}\right)}^{2}+\sum_{\forall i}\sum_{\forall j}2{\left({\left(k_{upd}^{er}\right)}_{ij}-{\left(k_{poi}^{er}\right)}_{ij}\right)}^{2}\right)$$

Subject to:

(23) $$t_{fus}^{od}={\left(X_{\mathrm{poi}}^{r}\right)}^{T}t_{\mathrm{upd}}^{r}+{\left(X_{\mathrm{poi}}^{e}\right)}^{T}t_{\mathrm{upd}}^{e,\ell -1}+{\left(X_{\mathrm{poi}}^{e}\right)}^{T}K_{\mathrm{upd}}^{\mathrm{er}}{\left(K_{\mathrm{poi}}^{\mathrm{rr}}\right)}^{-1}(t_{\mathrm{poi}}^{r}-t_{\mathrm{upd}}^{r,\ell -1})$$

(24) $${\left(\sigma_{fus}^{od}\right)}^{2}={\left(X_{\mathrm{poi}}^{r}\right)}^{T}K_{\mathrm{poi}}^{\mathrm{rr}}X_{\mathrm{poi}}^{r}+{\left(X_{\mathrm{poi}}^{e}\right)}^{T}K_{\mathrm{upd}}^{\mathrm{ee}}X_{\mathrm{poi}}^{e}+2{\left(X_{\mathrm{poi}}^{e}\right)}^{T}K_{\mathrm{upd}}^{\mathrm{er}}X_{\mathrm{poi}}^{r}$$

The nonlinear programming M1 has a convex objective function and two linear constraints. To ensure $K_{\mathrm{upd}}$ is stable over time, objective function (22) minimizes the total difference of updating elements in both $K_{\mathrm{upd}}^{\mathrm{ee}}$ and $K_{\mathrm{upd}}^{\mathrm{er}}$ sub-matrixes. Constraints (23) and (24), derived from Equations (9) and (10), ensure that the summation of means and variances of corresponding link travel time distributions are equal to that of the fused path travel time distribution, i.e., $t_{fus}^{od}$ and ${\left(\sigma_{fus}^{od}\right)}^{2}$. This M1 problem is a typical quadratic programming problem. A unique solution can be determined using several efficient algorithms, such as the quadprog function in MatLab.

Once $K_{\mathrm{upd}}$ is determined, the vector of travel time means for links without point detectors, $t_{\mathrm{upd}}^{e}$ are updated as:

(25) $$t_{\mathrm{upd}}^{e}=t_{\mathrm{upd}}^{e,\ell -1}+K_{\mathrm{upd}}^{\mathrm{er}}{\left(K_{\mathrm{poi}}^{\mathrm{rr}}\right)}^{-1}(t_{\mathrm{poi}}^{r}-t_{\mathrm{upd}}^{r,\ell -1})$$

The updated $t_{\mathrm{upd}}$ and $K_{\mathrm{upd}}$ are used for estimating travel time distributions of links without point detectors in the subsequent time interval. The detailed steps of the Algorithm 1 are summarized as follows.
**Algorithm 1**Step 1. Data preprocessing stage:Estimate $T_{int}^{od}$ from interval detector data at current interval ℓ.Estimate $t_{\mathrm{poi}}^{r}$ and $K_{\mathrm{poi}}^{\mathrm{rr}}$ for links with point detectors at current interval ℓ. Deduce $t_{\mathrm{poi}}^{e}$ and $v_{\mathrm{poi}}^{e}$ for links without point detectors using Equations (6) and (7). Estimate $T_{poi}^{od}$ using Equations (9) and (10).Step 2. Distribution fusion stage:Estimate $T_{fus}^{od}$ by fusing $T_{int}^{od}$ and based on Equations (11)–(21).Step 3. Posterior update stage:Update $K_{\mathrm{upd}}$ using Equations (22)–(24); and update $t_{\mathrm{upd}}$ using Equation (25).Set $K_{\mathrm{upd}}^{\ell -1}=K_{\mathrm{upd}}$, and $t_{\mathrm{upd}}^{\ell -1}=t_{\mathrm{upd}}$. Go to Step 1 for next time interval.


## 5. Numerical Experiments

**Figure 4:**
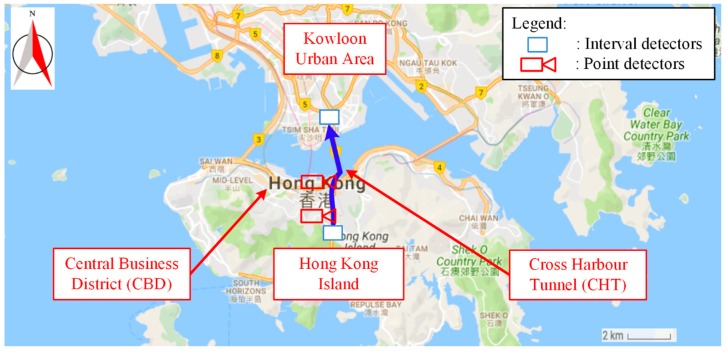
Study area location.

Performance of the proposed heterogeneous data fusion method was investigated using real-world data from Hong Kong, as shown in Figure 4. A path from Aberdeen tunnel in Hong Kong Island to the Cross Harbor tunnel (CHT) in Kowloon urban area was selected for this case study. CHT is the most congested of the three tunnels connecting Kowloon urban area and Hong Kong Island. The total travel distance of the chosen path was 3.7 km with free-flow travel time 3.6 min. There were 11 links in the chosen path, with only two, Links 1 and 5, equipped with Autoscope video image detectors (VIDs), which is a popular type of point detector. Two AVI devices were installed at the beginning and end of the chosen path for automatic toll collection. Market penetration of AVI systems was approximately 40%. Real-time AVI data were also utilized for the implementation of RTIS (real-time traveler information systems) in Hong Kong [14]. Detailed information of this AVI system was provided in Tam and Lam [14].

Traffic data from both interval and point detectors were collected during (07:00–23:00) of a typical weekday: Wednesday, 20 August 2014. An offline link travel time covariance matrix was obtained from RTIS [14] as the initial $K_{\mathrm{fus}}$. To evaluate the performance of the proposed heterogeneous data fusion method, a manual license plate matching survey was performed. Video recording equipment was set at the starting and end nodes of the chosen path to record the license plate readings of vehicles. The vehicles recorded at the starting and end nodes were manually matched. Path travel times of matched vehicles were computed as ground truth for accuracy validation.

### 5.1. Evaluation Metrics

Two widely accepted metrics, mean absolute percentage error (MAPE) and root mean square error (RMSE), were adopted to evaluate the accuracy of the estimated mean of path travel time distributions:

(26) $$MAPE_{t}=\frac{100\%}{n}\cdot \sum_{\ell =1}^{n}\frac{\left|t_{fus}^{od}-t_{obs}^{od}\right|}{t_{obs}^{od}}$$

(27) $$RMSE_{t}=\sqrt{\frac{1}{n}\cdot \sum_{\ell =1}^{n}{\left(t_{fus}^{od}-t_{obs}^{od}\right)}^{2}}$$

where *n* is the number of time intervals during the period of interest, and $t_{obs}^{od}$ is the ground truth observed mean travel time obtained from the field survey at time interval *ℓ*. Smaller *MAPE_t_* and *RMSE_t_* indicate higher accuracy of the estimated mean travel time.

The MAPE and RMSE concepts were extended to evaluate the accuracy of the estimate STD of the path travel time as:

(28) $$MAPE_{\sigma }=\frac{100\%}{n}\cdot \sum_{\ell =1}^{n}\frac{\left|\sigma_{fus}^{od}-\sigma_{obs}^{od}\right|}{\sigma_{obs}^{od}}$$

(29) $$RMSE_{\sigma }=\sqrt{\frac{1}{n}\cdot \sum_{\ell =1}^{n}{\left(\sigma_{fus}^{od}-\sigma_{obs}^{od}\right)}^{2}}$$

where $\sigma_{obs}^{od}$ represents the ground truth observed travel time STD obtained from the field survey at time interval *ℓ*.

For many transportation applications, it is meaningful to construct a travel time interval at a given confidence level from the estimated travel time distribution [60,61]. The travel time interval accuracy represents the integrated accuracy of both the estimated mean and STD. Two metrics were adopted to evaluate these accuracies: probability outside the predicted (estimated) time interval (POPI) and probability outside the observed time interval (POOI) [62]. The POPI measures the percentage of observed data outside the estimated travel time interval, while the POOI measures the percentage of estimated distribution outside the observed travel time interval.

Let $l_{fus}=\Phi_{fus}^{-1}(\alpha /2)$ and $u_{fus}=\Phi_{fus}^{-1}(1-\alpha /2)$ be the lower and upper bounds of the estimated travel time interval, respectively, at confidence level 1−α, where $\Phi_{fus}^{-1}(\cdot )$ is the inverse CDF of the estimated path travel time distribution. Then:

(30) $$POPI=\frac{100\%}{n}\cdot \sum_{\ell =1}^{n}\left(1-\frac{\Phi_{obs}(u_{fus})-\Phi_{obs}(l_{fus})}{1-\alpha }\right)$$

where $\Phi_{obs}(\cdot )$ is the CDF of the observed travel time distributions. The POPI value ranges from 0 to 1. The smaller POPI indicates capture of larger proportion of observed data, i.e., higher accuracy of the estimated travel time interval. As noted by Shi et al. [62], this POPI metric is very useful, but tends to exhibit bias for situations of wide travel time intervals due to large STD errors.

As an alternative, POOI metric is the percentage of estimated distribution outside the observed travel time interval. Let $l_{obs}=\Phi_{obs}^{-1}(\alpha /2)$ and $u_{obs}=\Phi_{obs}^{-1}(1-\alpha /2)$ denote the lower and upper bounds of the observed travel time interval, respectively, at confidence level 1−α, where $\Phi_{obs}^{-1}(\cdot )$ is the inverse CDF of the observed path travel time distribution, and $\Phi_{fus}(\cdot )$ denotes the CDF of the estimated travel time distribution. Then:

(31) $$POOI=\frac{100\%}{n}\cdot \sum_{\ell =1}^{n}\left(1-\frac{\Phi_{fus}(u_{obs})-\Phi_{fus}(l_{obs})}{1-\alpha }\right)$$

POOI also ranges [0, 1], and larger POOI indicates lower estimated travel time interval accuracy, because a larger proportion is outside the observed travel time interval. Therefore, the POPI and POOI matrices are complementary to evaluate the estimated path travel time distribution accuracy.

### 5.2. Experimental Results

This section reports experimental results of the case study using the proposed heterogeneous data fusion method. Travel time distributions for the chosen path and links were estimated every 2 min. The probability of the unknown state for both interval and point detectors was set as $\alpha_{\Theta }=m_{int}(\Theta )=m_{pos}(\Theta )=0.05$, and sensitivity parameters in Equations (15) and (16) were set as $\beta_{int}=0.2$ and $\beta_{poi}=0.8$, according to the sensitive analysis results obtained from Dion and Rakha [34]. Setting $\beta_{poi}=4\beta_{int}$ assigns a higher level of information quality to the interval detector than point detector data, given the same sample sizes.

**Figure 5:**
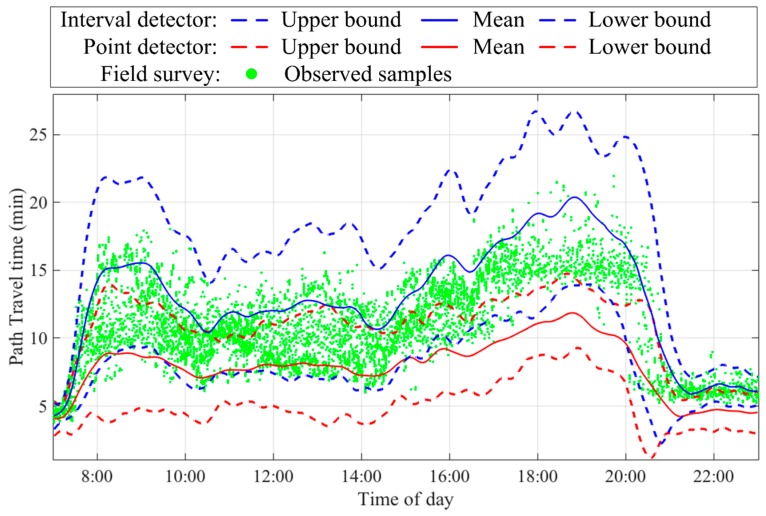
Two path travel time distributions obtained in the data preprocessing step.

Figure 5 shows two path travel time distributions, $T_{int}^{od}$ and $T_{poi}^{od}$, estimated from interval and point detectors, respectively, in the data preprocessing step. Travel time intervals were constructed for the 95% confidence level, i.e., $\alpha_{\Theta }=0.05$, for both interval and point detectors, shown in blue and red, respectively. Observed data from the field survey, shown in green dots, were only used for accuracy validation. As shown in the figure, two estimated travel time intervals from different data sources can cover most observed data well during the period of interest. The two estimated travel time distributions show high consistency during off-peak periods (21:00–23:00 and 7:00–7:30), slight inconsistency during inter-peak periods (10:00–16:00), and high inconsistency during peak periods (7:30–10:00 and 16:00–21:00). In general, $T_{int}^{od}$ tended to have higher accuracy than $T_{poi}^{od}$. This was expected, since $T_{int}^{od}$ was estimated from interval detector data, whereas $T_{poi}^{od}$ was estimated from point detector data through spatial interpolation. Such a result also justified the chosen sensitivity parameters, reflecting the higher level of information quality for the interval detector data.

**Figure 6:**
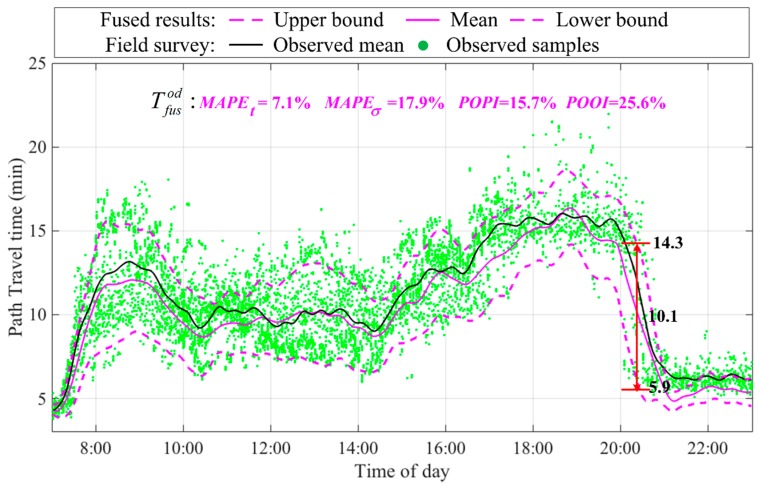
Fused path travel time distribution during the period of interest.

Figure 6 shows the resultant path travel time distribution after fusing the two path travel time distributions from Figure 5. A confidence level of 80%, i.e., *α* = 0.2, was used to construct the travel time interval and calculate POPI and POOI metrics. The proposed heterogeneous data fusion method provided an accurate and robust estimation of mean travel time, $t_{fus}^{od}$, throughout the period of interest, with $MAPE_{t}=7.1\%$. However, the relative large $MAPE_{\sigma }=17.9\%$ showed that the proposed method overestimated path travel time distribution STD, $\sigma_{fus}^{od}$, for the period of interest. This highlights the challenge of accurately estimating $\sigma_{fus}^{od}$ in congested road networks. One major reason may be the difficulty of estimating $\sigma_{obs}^{od}$ of the population using biased samples with various data quality. Fortunately, the slight STD over estimation could be beneficial to most travelers with risk-averse attitudes regarding travel time uncertainty. POPI=15.7%, somewhat better than the target (20%), which indicates that a high proportion (84.3%) of observation data was well covered by the estimated path travel time interval. It can also be seen from the figure that the estimated interval was not too wide, given the relative large STD error. POOI=25.6%, which was somewhat larger than the target (20%). Thus, overall the POPI and POOI metrics verified that the proposed heterogeneous data fusion method could obtain accurate and robust estimations of the path travel time interval (i.e., path travel time distribution) by fusing heterogeneous interval and point detector data.

### 5.3. Comparison of Data Fusion and Single Data Source Results

**Figure 7:**
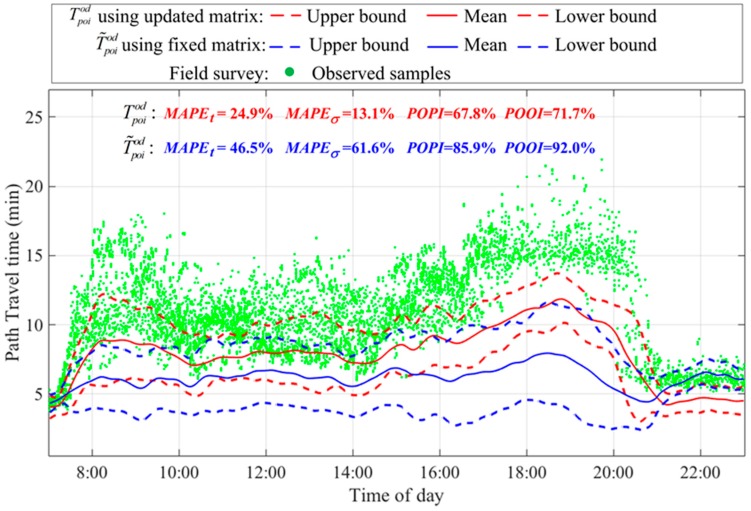
Path travel time distributions estimated from point detector data by using updated and fixed spatial correlations.

In this section, the effectiveness of the proposed heterogeneous data fusion method was investigated by comparing data fusion results with those estimated from single data source. The estimated path travel time distribution (i.e., $T_{int}^{od}$) from single interval detector data was shown in Figure 5 in blue. The estimated path travel time distribution from single point detector data (denoted by ${\tilde{T}}_{poi}^{od}$) was shown in Figure 7 in blue, which was different from the $T_{poi}^{od}$ estimation shown in Figure 5. It should be noted that ${\tilde{T}}_{poi}^{od}$ was generated using fixed offline spatial correlations obtained from RTIS, and $T_{poi}^{od}$ was generated by the proposed heterogeneous data fusion method using the updated spatial correlations.

Figure 7 shows travel time intervals of ${\tilde{T}}_{poi}^{od}$ and $T_{poi}^{od}$ in blue and red colors for comparison. The 80% confidence level was used for construing travel time intervals and calculating POPI and POOI metrics. As illustrated, by using updated spatial correlations, the accuracy of the path travel time distribution estimated from point detector data was significantly improved. The $MAPE_{t}$, $MAPE_{\sigma }$, POPI, and POOI metrics were reduced by 46.4% (i.e., 1–24.9%/46.5%), 78.9%, 21.1%, and 22.1%, respectively. This validates the effectiveness of the proposed optimization technique for updating travel time spatial correlations. Such a result also highlights the necessity for considering the dynamic nature of travel time spatial correlations in congested road networks, and implies that current spatial interpolation techniques [14,15] built on fixed spatial correlations may lead to considerable errors when imputing missing data.

Table 3 summarizes the evaluation metrics for all path travel time distributions estimated from point detector, ${\tilde{T}}_{poi}^{od}$; interval detector, $T_{int}^{od}$; and fused data, $T_{fus}^{od}$. Amongst these three distributions, the accuracy of ${\tilde{T}}_{poi}^{od}$ was the poorest, with $MAPE_{t}=46.5\%$ and $MAPE_{\sigma }=61.6\%$. The POPI=85.9% indicates that a large proportion (85.9%) of observations falling outside the travel time interval of ${\tilde{T}}_{poi}^{od}$. The POOI=92.0% shows that almost whole travel time range of ${\tilde{T}}_{poi}^{od}$ was out of the observed time interval. The accuracy of $T_{int}^{od}$ was somewhat superior, with $MAPE_{t}=17.1\%$, $MAPE_{\sigma }=76.9\%$, POPI=26.4% and POOI=48.9%. As shown, $T_{fus}^{od}$, using the proposed data fusion method, was the best for all evaluation metrics. By fusing interval and point detector data, the $MAPE_{t}$, $MAPE_{\sigma }$, POPI and POOI metrics were respectively reduced by 58.5% (i.e., 1–7.1%/17.1%), 76.7%, 40.5%, and 47.6%, when compared to that of $T_{int}^{od}$. Thus, the proposed heterogeneous data fusion method can significantly improve the accuracy of path travel time distribution estimations from interval and point detectors.

**Figure 8:**
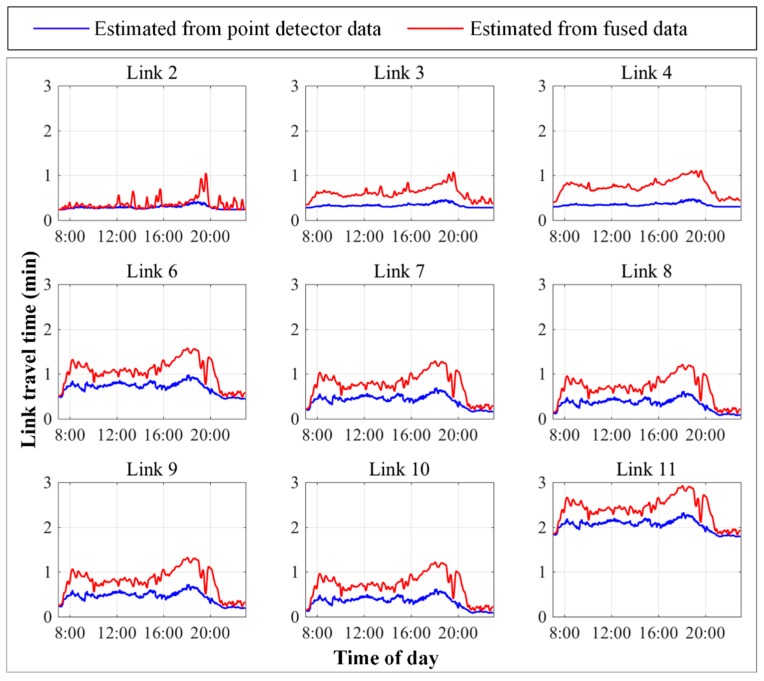
Individual link travel time distribution estimated from point detector data and fused data.

Fusion of interval and point detector data can improve the accuracy of travel time distributions for links without point detectors. When only point detector data were used, travel time distributions for links without point detectors were indirectly estimated through the fixed spatial correlations. Fusing interval and point detector data provided better estimations of link travel time distributions from the updated spatial correlations. Figure 8 compares individual link travel time distributions estimated from point detector data and the proposed data fusion method. Ground truth data for these link travel time distributions were not available for quantitative analysis of estimation accuracy. Nevertheless, link travel time distributions estimated from the proposed heterogeneous data fusion method better capture dynamic traffic conditions, with more distinct peaks occurring during the morning and evening peak periods. The much superior accuracy of path travel time distribution estimation (see Table 3) also justifies this visual observation, because the path travel time distribution is the summation of corresponding link travel time distributions along the path.

### 5.4. Comparison of Different Distribution Fusion Algorithms

This section investigates the effectiveness of the proposed D-S distribution fusion algorithm built on the D-S evidence theory. To further evaluate and benchmark the proposed algorithm, a linear combination fusion algorithm built on the linear combination approach was also implemented. The linear combination approach (or simple convex combination approach) has been widely used as a simple and effective technique to fuse two independent estimations of mean travel times [11],

(32) $$t_{fus}^{od}=\frac{w_{int}}{w_{int}+w_{poi}}t_{int}^{od}+\frac{w_{poi}}{w_{int}+w_{poi}}t_{poi}^{od}$$

where $w_{int}$ and $w_{poi}$ are the data quality of interval and point detectors, respectively, as defined in Equations (15) and (16). This study extended the linear combination approach to fuse two independent STD estimations, as:

(33) $$\sigma_{fus}^{od}=\frac{w_{int}}{w_{int}+w_{poi}}\sigma_{int}^{od}+\frac{w_{poi}}{w_{int}+w_{poi}}\sigma_{poi}^{od}$$

Assuming normal distributions, this extended linear combination fusion algorithm can be used to fuse path travel time distributions from interval and point detectors.

**Figure 9:**
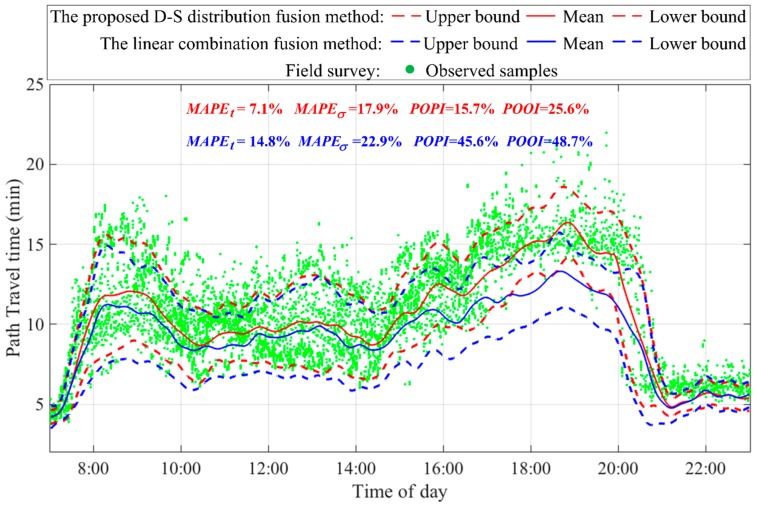
Path travel times of different methods during the period of interest.

In this study, the same set of input data was used to validate the results of the proposed D-S distribution fusion and the linear combination fusion algorithms. Path travel time distributions of interval and point detectors obtained in the data preprocessing step, shown in Figure 5, were employed as the input data. Figure 9 reports the fused path travel time distributions using these two algorithms. As shown, the proposed D-S distribution fusion algorithm produces better of path travel time distribution estimates than the linear combination fusion algorithm. The proposed algorithm can significantly reduce $MAPE_{t}$, $MAPE_{\sigma }$, POPI, and POOI metrics by 58.6%, 15.3%, 37.2%, and 38.0%, respectively, compared to the linear combination fusion algorithm. This result indicates that the D-S evidence theory is effective for fusing inaccurate and inconsistent distribution data from multiple sources under various information conflict situations, including highly consistent, slightly inconsistent, and highly inconsistent situations.

## 6. Conclusions and Future Research

Provision of travel time distribution information is a crucial requirement for travelers to make reliable path choice decisions incorporating travel time uncertainties. With advances in information and communication technologies, interval detectors (such as automatic vehicle identification devices) and point detectors (such as loop detectors) are being increasingly deployed in road networks. These interval and point detectors generate heterogeneous data sources with distinct characteristics of data quality and network coverage. Fusing these heterogeneous data can be beneficial for robust and accurate estimation of travel time distribution information.

This paper proposed a heterogeneous data fusion method to estimate travel time distributions, fusing heterogeneous data from point and interval detectors. The proposed method consisted of three steps. The first step, i.e., data preprocessing, was to respectively estimate path travel time distributions from interval and point detector data. The spatially missing data issue of point detectors was addressed. The travel time distributions of links without point detectors were imputed based on their spatial correlations with links that had point detectors. The second step, i.e., distribution fusion, was to fuse these two path travel time distributions estimated from interval and point detectors. A D-S distribution fusion algorithm built on the Dempster-Shafer evidence theory was proposed to fuse path travel time distributions from different data sources with various information qualities. The third step, i.e., posterior update, was to update link travel time distributions and their spatial correlations. The problem of updating spatial correlations was formulated and solved as a quadratic programming problem with a convex objective function and two linear constraints.

To validate the accuracy of the proposed heterogeneous data fusion method, a case study was performed using real-world data from RTIS in Hong Kong. The results validated that the proposed method can obtain robust and accurate estimations of path travel time distributions in congested road networks. Compared with either interval or point detectors alone, the proposed data fusion method can significantly reduce estimation errors for path travel time distributions with respect to $MAPE_{t}$, $MAPE_{\sigma }$, POPI, and POOI metrics. The proposed D-S distribution fusion algorithm was also compared to a linear combination algorithm for the same case study, and it showed that the proposed D-S distribution fusion algorithm can generate a robust and accurate fusion of travel time distributions over the whole period of interest, including highly consistent, slightly inconsistent, and highly inconsistent situations for the different data sources. Furthermore, the results of the case study indicated that the proposed optimization technique can effectively update travel time spatial correlations under dynamic traffic conditions, and incorporation of updated spatial correlations greatly enhanced estimation accuracy of travel time distributions of the path and all links without point detectors. Therefore, the proposed D-S distribution algorithm was validated to be effective for fusing travel time distributions from different data sources under various information conflict situations, including highly consistent, slightly inconsistent, and highly inconsistent situations.

There are several worthwhile directions for future research. First, travel times in this study were assumed to follow normal distributions. However, several previous studies have found that travel times in congested road networks could be better represented by asymmetric distributions with strong positive skew, e.g., lognormal, gamma, or Burr distributions [10,57]. The proposed heterogeneous data fusion method can be easily extended to other types of distributions with two parameters, e.g., lognormal or gamma, by replacing Equation (14) with corresponding methods to calculate the cumulative distribution function. Second, the spatial interpolation proposed by Tam and Lam [14] was adopted in this study for imputing the travel time distributions of links without point detectors. However, other effective spatial interpolation techniques have been proposed, such as Kriging [15]. Integrating these alternative spatial interpolation techniques into the proposed heterogeneous data fusion method warrants further study. Third, the proposed data fusion method only considered heterogeneous data from point and interval detectors. How to extend the proposed method to incorporate floating car data needs further investigation. Fourth, the case study only involved a specific path. Extension of the proposed method to fuse travel time distributions of multiple paths between a pair of nodes is an interesting topic for further investigation. Last but not the least, travel time distributions were estimated in this study for the current time interval. Extension of the proposed data fusion method to the problem of short term travel time distribution prediction is another interesting topic for further study.

## Figures and Tables

**Table 1:** Simple example of distribution fusion using Dempster’s combination rule.

Travel Time Ranges	Case 1			Case 2			Case 3		
	$m_{int}(\cdot )$	$m_{poi}(\cdot )$	$m_{fus}(\cdot )$	$m_{int}(\cdot )$	$m_{poi}(\cdot )$	$m_{fus}(\cdot )$	$m_{int}(\cdot )$	$m_{poi}(\cdot )$	$m_{fus}(\cdot )$
$S_{1}$	0.1	0	0	0.3	0	0	0.4	0	-
$S_{2}$	0.2	0.3	0.2143	0.6	0	0	0.6	0	-
$S_{3}$	0.4	0.4	**0.5714**	0.1	0.1	**1**	0	0	-
$S_{4}$	0.2	0.3	0.2143	0	0.6	0	0	0.7	-
$S_{5}$	0.1	0	0	0	0.3	0	0	0.3	-

**Table 2:** Simple example of distribution fusion using the generalized combination rule.

Travel Time Ranges	Case 1			Case 2			Case 3		
	$m_{int}(\cdot )$	$m_{poi}(\cdot )$	$m_{fus}(\cdot )$	$m_{int}(\cdot )$	$m_{poi}(\cdot )$	$m_{fus}(\cdot )$	$m_{int}(\cdot )$	$m_{poi}(\cdot )$	$m_{fus}(\cdot )$
$S_{1}$	0.075	0	0.0410	0.275	0	0.2415	0.375	0	0.3337
$S_{2}$	0.2	0.275	0.2075	0.6	0	0.5270	0.575	0	0.5116
$S_{3}$	0.4	0.4	0.4756	0.075	0.075	**0.0874**	0	0	0.0000
$S_{4}$	0.2	0.275	0.2075	0	0.6	0.0687	0	0.675	0.0783
$S_{5}$	0.075	0	0.0410	0	0.275	0.0315	0	0.275	0.0319
Θ	0.05	0.05	0.0273	0.05	0.05	0.0439	0.05	0.05	0.0445

**Table 3:** The accuracy of data fusion results and single data source results.

Data Source	Estimated Mean		Estimated STD		*POPI*	*POOI*
	MAPE	RMSE (min)	MAPE	RMSE (min)		
Point detectors	46.5%	2.32	61.6%	0.75	85.9%	92.0%
Interval detectors	17.1%	1.42	76.9%	1.01	26.4%	48.9%
Data fusion	7.1%	0.85	17.9%	0.35	15.7%	25.6%

## References

[1] Chen B.Y., Lam W.H.K., Sumalee A., Li Q.Q., Shao H., Fang Z.X. (2013). Finding reliable shortest paths in road networks under uncertainty. Netw. Spat. Econ..

[2] Chen B.Y., Li Q.Q., Lam W.H.K. (2016). Finding the k reliable shortest paths under travel time uncertainty. Transp. Res. B Methodol..

[3] Yang L., Zhou X. (2017). Optimizing on-time arrival probability and percentile travel time for elementary path finding in time-dependent transportation networks: Linear mixed integer programming reformulations. Transp. Res. B Methodol..

[4] Zhong R., Sumalee A., Maruyama T. (2012). Dynamic marginal cost, access control, and pollution charge: A comparison of bottleneck and whole link models. J. Adv. Transp..

[5] Zhao J., Ma W., Liu Y., Han K. (2016). Optimal operation of freeway weaving segment with combination of lane assignment and on-ramp signal control. Transp. A.

[6] Chen B.Y., Yuan H., Li Q.Q., Shaw S.L., Lam W.H.K., Chen X. (2016). Spatiotemporal data model for network time geographic analysis in the era of big data. Int. J. Geogr. Inf. Sci..

[7] Lim S., Lee C. (2011). Data fusion algorithm improves travel time predictions. IET Intell. Transp. Syst..

[8] Mori U., Mendiburu A., Alvarez M., Lozano J.A. (2015). A review of travel time estimation and forecasting for Advanced Traveller Information Systems. Transp. A.

[9] Chen B.Y., Yuan H., Li Q.Q., Lam W.H.K., Shaw S.L., Yan K. (2014). Map matching algorithm for large-scale low-frequency floating car data. Int. J. Geogr. Inf. Sci..

[10] Du L., Peeta S., Kim Y.H. (2012). An adaptive information fusion model to predict the short-term link travel time distribution in dynamic traffic networks. Transp. Res. B Methodol..

[11] Bachmann C., Abdulhai B., Roorda M.J., Moshiri B. (2013). A comparative assessment of multi-sensor data fusion techniques for freeway traffic speed estimation using microsimulation modeling. Transp. Res. C Emerg. Technol..

[12] Bachmann C., Roorda M.J., Abdulhai B., Moshiri B. (2013). Fusing a bluetooth traffic monitoring system with loop detector data for improved freeway traffic speed estimation. J. Intell. Transp. Syst..

[13] Deng W., Lei H., Zhou X. (2013). Traffic state estimation and uncertainty quantification based on heterogeneous data sources: A three detector approach. Transp. Res. B Methodol..

[14] Tam M.L., Lam W.H.K. (2008). Using automatic vehicle identification data for travel time estimation in Hong Kong. Transportmetrica.

[15] Zou H., Yue Y., Li Q., Yeh A.G.O. (2012). An improved distance metric for the interpolation of link-based traffic data using kriging: A case study of a large-scale urban road network. Int. J. Geogr. Inf. Sci..

[16] El Esawey M., Sayed T. (2011). Travel time estimation in urban networks using limited probes data. Can. J. Civil. Eng..

[17] Liu H.X., Ma W. (2009). A virtual vehicle probe model for time-dependent travel time estimation on signalized arterials. Transp. Res. C Emerg. Technol..

[18] Liu H.X., Ma W., Wu X., Hu H. (2012). Real-time estimation of arterial travel time under congested conditions. Transportmetrica.

[19] Ndoye M., Totten V.F., Krogmeier J.V., Bullock D.M. (2011). Sensing and signal processing for vehicle reidentification and travel time estimation. IEEE Trans. Intell. Transp. Syst..

[20] Yu B., Lam W.H.K., Tam M.L. (2011). Bus arrival time prediction at bus stop with multiple routes. Transp. Res. C Emerg. Technol..

[21] Vlahogianni E.I., Golias J.C., Karlaftis M.G. (2004). Short-term traffic forecasting: Overview of objectives and methods. Transp. Rev..

[22] El Faouzi N.E. Data fusion in road traffic engineering: An overview. Proceedings of the SPIE—The International Society for Optical Engineering.

[23] Choi K., Chung Y. (2002). A data fusion algorithm for estimating link travel time. J. Intell. Transp. Syst..

[24] El Faouzi N.E. Bayesian and evidential approaches for traffic data fusion: Methodological issues and case study. Proceedings of the Transportation Research Board 85th Annual Meeting (No. 06–1510).

[25] El Faouzi N.E., Klein L.A., De Mouzon O. (2009). Improving travel time estimates from inductive loop and toll collection data with Dempster-Shafer data fusion. Transport. Res. Rec..

[26] Kong Q.J., Li Z., Chen Y., Liu Y. (2009). An approach to urban traffic state estimation by fusing multisource information. IEEE Trans. Intell. Transp. Syst..

[27] Kong Q.J., Chen Y., Liu Y. (2009). A fusion-based system for road-network traffic state surveillance: A case study of Shanghai. IEEE Intell. Transp. Syst..

[28] Nantes A., Dong N., Bhaskar A., Miska M., Chung E. (2015). Real-time traffic state estimation in urban corridors from heterogeneous data. Transp. Res. C Emerg. Technol..

[29] Shan Z., Xia Y., Hou P., He J. (2016). Fusing Incomplete Multisensor Heterogeneous Data to Estimate Urban Traffic. IEEE Multimed..

[30] Lederman R., Wynter L. (2011). Real-time traffic estimation using data expansion. Transp. Res. B Methodol..

[31] Haworth J., Cheng T. (2012). Non-parametric regression for space-time forecasting under missing data. Comput. Environ. Urban..

[32] Li L., Li Y.B., Li Z.H. (2013). Efficient missing data imputing for traffic flow by considering temporal and spatial dependence. Transp. Res. C Emerg. Technol..

[33] Chan K.S., Lam W.H.K., Tam M.L. (2009). Real-time estimation of arterial travel times with spatial travel time covariance relationships. Transp. Res. Rec..

[34] Dion F., Rakha H. (2006). Estimating dynamic roadway travel times using automatic vehicle identification data for low sampling rates. Transp. Res. B Methodol..

[35] Jenelius E., Koutsopoulos H.N. (2013). Travel time estimation for urban road networks using low frequency probe vehicle data. Transp. Res. B Methodol..

[36] Rahmani M., Jenelius E., Koutsopoulos H.N. (2015). Non-parametric estimation of route travel time distributions from low-frequency floating car data. Transp. Res. C Emerg. Technol..

[37] Hans E., Chiabaut N., Leclercq L. (2015). Applying variational theory to travel time estimation on urban arterials. Transp. Res. B Methodol..

[38] Dempster A.P. (1967). Upper and lower probabilities induced by multi-valued mapping. Ann. Math. Stat..

[39] Shafer G. (1976). A Mathematical Theory of Evidence.

[40] Beynon M., Cosker D., Marshall D. (2001). An expert system for multi-criteria decision making using Dempster Shafer theory. Expert Syst. Appl..

[41] Hegarat-Mascle S.L., Richard D., Ottle C. (2003). Multi-scale data fusion using Dempster-Shafer evidence theory. Integr. Comput. Aided Eng..

[42] Gong Y., Wang Y. Application Research on Bayesian Network and D-S Evidence Theory in Motor Fault Diagnosis. Proceedings of the 6th International Conference on Intelligent Networks and Intelligent Systems (ICINIS).

[43] Khaleghi B., Khamis A., Karray F.O., Razavi S.N. (2013). Multisensor data fusion: A review of the state-of-the-art. Inf. Fusion.

[44] Deng Y., Chan F.T.S. (2011). A new fuzzy dempster MCDM method and its application in supplier selection. Expert Syst. Appl..

[45] Su S.Y., Deng Y., Mahadevan S., Bao Q.L. (2012). An improved method for risk evaluation in failure modes and effects analysis of aircraft engine rotor blades. Eng. Fail. Anal..

[46] Parikh C.R., Pont M.J., Jones N.B. (2001). Application of Dempster–Shafer theory in condition monitoring applications: A case study. Pattern Recogn. Lett..

[47] Dou Z., Xu X., Lin Y., Zhou R. (2014). Application of D-S evidence fusion method in the fault detection of temperature sensor. Math. Probl. Eng..

[48] Fan X., Zuo M.J. (2006). Fault diagnosis of machines based on D-S evidence theory. Part 1: D–S evidence theory and its improvement. Pattern Recogn. Lett..

[49] Hu Y., Fan X., Zhao H., Hu B. The Research of Target Identification Based on Neural Network and D-S Evidence Theory. Proceedings of the International Asia Conference on Informatics in Control.

[50] Dong G., Kuang G. (2015). Target recognition via information aggregation through Dempster–Shafer’s evidence theory. IEEE Geosci. Remote Sens..

[51] Li Y., Chen J., Ye F., Liu D. (2016). The improvement of DS evidence theory and its application in IR/MMW target recognition. J. Sens..

[52] Dymova L., Sevastjanov P. (2010). An Interpretation of Intuitionistic Fuzzy Sets in the Framework of the Dempster-Shafer Theory: Decision making aspect. Knowl. Based Syst..

[53] Chen N., Sun F., Ding L., Wang H. (2009). An adaptive PNN-DS approach to classification using multi-sensor information fusion. Neural Comput. Appl..

[54] Yager R.R. (1987). On the Dempster-Shafer framework and new combination rules. Inf. Sci..

[55] Smets P. (1990). The Combination of Evidence in the Transferable Belief Model. IEEE Trans. Pattern Anal..

[56] Murphy C.K. (2000). Combining belief functions when evidence conflicts. Decis. Support Syst..

[57] Chen B.Y., Shi C., Zhang J., Lam W.H.K., Li Q.Q., Xiang S. (2016). Most reliable path-finding algorithm for maximizing on-time arrival probability. Transp. B.

[58] Lomax T., Schrank D., Turner S., Margiotta R. (2003). Selecting Travel Reliability Measures.

[59] Hart R.G. (1966). A Close approximation related to the error function. Math. Comput..

[60] Khosravi A., Mazloumi E., Nahavandi S., Creighton D., Van Lint J.W.C. (2011). A genetic algorithm-based method for improving quality of travel time prediction intervals. Transp. Res. C Emerg. Technol..

[61] Khosravi A., Mazloumi E., Nahavandi S., Creighton D., Van Lint J.W.C. (2011). Prediction intervals to account for uncertainties in travel time prediction. IEEE Trans. Intell. Transp. Syst..

[62] Shi C., Chen B.Y., Li Q. (2017). Estimation of Travel Time Distributions in Urban Road Networks Using Low-Frequency Floating Car Data. ISPRS Int. J. Geo-Inf..

